# Artificial Intelligence-Based MRI Segmentation in Glioblastoma and Single Brain Metastasis: An Exploratory Study of Diagnostic and Prognostic Value

**DOI:** 10.3390/life16050779

**Published:** 2026-05-07

**Authors:** Costin Chirica, Oriana-Maria Onicescu, Daniela Pomohaci, Mihai Ștefan Cristian Haba, Sabina-Ioana Chirica, Sergiu-Andrei Dinu, Laura-Elena Cucu, Gabriela Florența Dumitrescu, Maria Magdalena Leon, Danisia Haba

**Affiliations:** 1 Grigore T. Popa University of Medicine and Pharmacy Iasi, 700115 Iasi, Romania; 2 Faculty of Computer Science, Alexandru Ioan Cuza University Iasi, 700506 Iasi, Romania; 3 Department of Pathology, Prof. Dr. Nicolae Oblu Emergency Clinical Hospital Iasi, 700309 Iasi, Romania

**Keywords:** glioblastoma, single brain metastasis, artificial intelligence, tumor segmentation, machine learning, deep learning, overall survival

## Abstract

(1) Background: Differentiating between glioblastoma (GB) and single brain metastasis (SBM) on conventional MRI remains a clinical challenge, and manual tumor volumetry often fails to provide a robust prognostic signal for survival. This study evaluates whether AI-based automated segmentation and the derivation of multi-compartment volumetric ratios can improve diagnostic accuracy and survival prediction. (2) Methods: A retrospective study was conducted on 123 patients (*n* = 84 GB; *n* = 39 SBM) who underwent 1.5 T MRI. Automated segmentation was performed using a CE-certified deep learning tool to quantify total tumor, contrast-enhancing, necrotic, and peritumoral edema volumes. AI volumes were validated against the manual ellipsoidal method estimates. Three machine learning (ML) models were developed to predict 6-month overall survival: Model 1 (manual volumetry), Model 2 (AI sub-compartment volumes), and Model 3 (enhanced feature model with normalized ratios). (3) Results: AI-derived total volumes showed excellent correlation with manual measurements (r = 0.955, *p* < 0.001). GB exhibited a significantly higher AI Model Necrosis Volume/AI Model Total Volume Ratio (*p* < 0.001), while SBM showed a higher AI Model Edema Volume/AI Model Total Volume Ratio (*p* = 0.003). In survival tasks, the ML enhanced feature model showed improved discrimination over manual methods, reaching an AUC-ROC of 0.783 compared to 0.571 for manual volumetry—a +21.2% improvement. AI Model Contrast Volume/AI Model Total Volume Ratio and AI Model Edema Volume/AI Model Total Volume Ratio were identified as the leading prognostic predictors within this exploratory cohort. (4) Conclusions: In this exploratory analysis, AI-assisted multi-compartment segmentation appears to provide granular, biologically relevant imaging biomarkers that may complement traditional manual volumetry in the prognostic assessment of GB and SBM. Given the retrospective design and the absence of molecular profiling, these preliminary findings should be interpreted with caution and warrant prospective, multicenter validation.

## 1. Introduction

Intracranial tumoral pathology is characterized by significant heterogeneity, spanning a broad biological spectrum that ranges from well-differentiated benign lesions to highly aggressive types of cancerous tumors [1,2,3,4].

In regard to malignant masses, glioblastoma (GB) stands as the most prevalent primary malignant brain tumor in adults. This distinct biological entity arises from glial cells and is characterized by its notorious aggressiveness and high recurrence rate, resulting in a median overall survival (OS) of approximately 14.6 months despite multimodal therapy [5]. On the other hand, single brain metastasis (SBM) originating from extracranial primary malignancies exhibits not only a distinct etiology, but also a different clinical progression, necessitating tailored therapeutic protocols specific to the nature of the primary tumor [6]. SBM is typically localized at the gray-white matter junction. However, intracranial secondary involvement may represent the initial manifestation of an occult malignancy, necessitating a comprehensive search for the primary site (e.g., lung cancer, breast cancer, melanoma, renal cell carcinoma, or colorectal cancer) [7,8]. Accordingly, the extant literature highlights significant disparities between the epidemiological profiles of GB and those of SBM (see Table 1).

The differential diagnosis between GB and SBM in patients lacking a documented oncological record remains a significant diagnostic challenge. Nonetheless, it represents a prerequisite for rigorous clinical management. In this context, the initial imaging characterization of the lesion is imperative, as it provides critical insight into a presumptive diagnosis. Heightened clinical suspicion can dictate the primary surgical intent, ranging from a minimally invasive biopsy to a radical resection with considerable post-operative morbidity. Nonetheless, the initial neuroimaging findings must be further validated by histopathological assessment, which implies a rigorous analysis of tumor architecture and cytological features. This step-by-step approach is required in order to establish a definitive diagnosis. Thus, surgical intervention—whether through biopsy or resection—yields the requisite specimens for the final determination of the histological subtype, grading, and molecular profiling, all of which are parameters indispensable for a personalized therapeutic stratification [24,25].

In light of these aforementioned considerations, pre- and post-contrast brain magnetic resonance imaging (MRI) serves as the gold-standard modality for the initial detection of GB and SBM (while histopathology remains the gold standard for a definitive diagnosis), enabling a precise radiological characterization of the lesions’ localization, volumetry, and their respective spatial relationships with contiguous neural structures. Moreover, contrast enhancement dynamics provide critical insights into tumor biology, its invasive potential, and angiogenic profile, going beyond basic anatomical evaluation. For instance, robust and rapid contrast uptake indicates a substantial disruption of the blood–brain barrier and an elevated microvascular density, both of which are hallmarks of malignancy [26,27]. However, while MRI may detect and provide a preliminary indication of specific tumor types, histological examination provides the unequivocal differentiation of the tumoral subtypes, which exhibit distinct prognostic and therapeutic implications. Thus, establishing a definitive diagnosis facilitates personalized management tailored to the molecular and pathological profile of each case [28].

Furthermore, these two categories of malignant cerebral neoplasms must be differentiated in order to facilitate consequent targeted therapeutic strategies. Primary malignant tumors require a comprehensive approach, which may include maximal surgical resection with safe margins, radiotherapy, and chemotherapy, tailored to the histological subtype. On the other hand, secondary brain involvement may benefit from local treatment (radiosurgery, radiotherapy) combined with systemic therapy directed against the primary tumor. This differentiation in tumor management is crucial for patient prognosis and survival, as brain metastases typically reflect an advanced systemic disease [29].

Nowadays, there is an urgent clinical need for more objective, quantitative tools to enhance the radiological diagnostic precision [30,31]. The integration of AI into medical imaging has emerged as a transformative paradigm. Specifically, AI-driven segmentation—leveraging deep learning (DL) architectures—allows for the precise, automated delineation of tumor volumes and the extraction of high-dimensional, quantitative features that are often imperceptible to the human eye [32]. The traditional manual segmentation of brain tumors is a labor-intensive process, inherently susceptible to inter-observer variability and subjective interpretation. In this landscape, AI, particularly DL architectures such as Convolutional Neural Networks (CNNs), offers an innovative approach by providing automated, replicable, and highly precise volumetric assessments. These algorithms can delineate distinct intratumoral regions—including the necrotic core, the contrast-enhancing rim, and the peritumoral edema—with a level of granularity that often transcends conventional radiological evaluation [33,34,35].

Recent literature suggests that the primary advantage of the AI-driven approach lies in its ability to transform qualitative imaging findings into defined volumetric parameters. These quantitative indices—such as the total tumor volume and the relative ratios between different tumoral compartments—can then be directly correlated with patient survival outcomes [36,37]. This objective volumetric assessment serves as a robust prognostic framework, facilitating a systematic evaluation of how tumor burden and peritumoral edema extent influence OS. Specifically, AI-driven volumetric quantification of these compartments identifies objective biomarkers that may correlate with OS in both GB and SBM patients, thereby offering superior prognostic accuracy. Despite this prognostic potential, the application of such methodologies to differential diagnosis remains sparse and yields inconsistent results [6,38].

The aim of our research is to explore the potential of AI-based segmentation applied to MRI in differentiating GB from SBM, while also establishing correlations with patient survival. The present study aims to address this gap by evaluating whether a small set of biologically interpretable volumetric ratios—derived from a CE-certified, clinically deployable segmentation tool—can simultaneously support GB versus SBM differentiation and 6-month survival stratification, and by quantifying the incremental diagnostic and prognostic value of these ratios over both manual ellipsoidal volumetry and absolute AI-derived sub-compartment volumes within a unified machine learning framework.

## 2. Materials and Methods

### 2.1. Study Design and Ethical Approval

This study was conducted as a retrospective observational study, enrolling patients diagnosed with either GB or SBM at the Clinical Emergency Hospital Prof. Dr. Nicolae Oblu Iasi, Romania, between January 2019 and April 2024. To ensure the consistency of survival data, only patients who followed standardized institutional treatment protocols (maximal safe resection followed by the Stupp protocol for GB, and surgical resection or stereotactic radiosurgery for SBM) were included [39].

The study was conducted in accordance with the principles of the Declaration of Helsinki and received approval from the Ethics Committee of the Grigore T. Popa University of Medicine and Pharmacy Iasi (No. 421/31 March 2024 for GB patients and No. 422/2 April 2024 for SBM patients), as well as from the Ethics Committee of the Clinical Emergency Hospital “Prof. Dr. Nicolae Oblu” (No. 10/8 November 2023).

**Figure 1:**
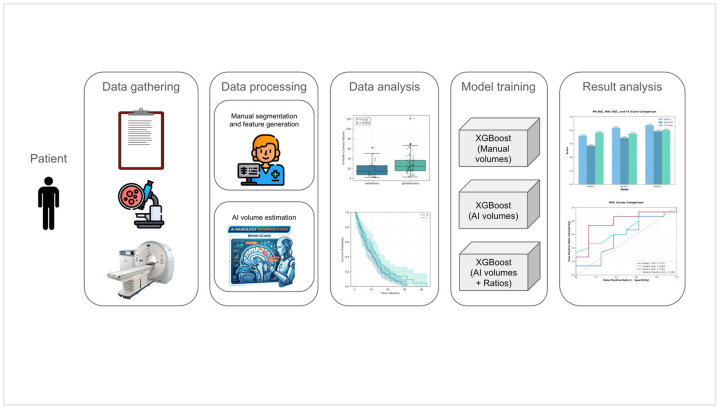
A flowchart summarizing the complete analytical pipeline of the study.

An overview of the complete study workflow, including MRI data processing, AI-based segmentation, data analysis, model training, and result analyses, is presented in Figure 1.

### 2.2. Patient Selection

A total of 123 patients were enrolled, comprising 84 individuals with histopathologically confirmed GB and 39 with histopathologically confirmed SBM.

Eligibility for this study was restricted to adult patients, aged eighteen years or older at the time of diagnosis, who underwent a complete pre- and post-contrast brain MRI examination according to the standardized institutional imaging protocol. All participants required histopathologically confirmed diagnoses of either GB or SBM, established through surgical resection or stereotactic biopsy. To maintain diagnostic homogeneity across the study period, all cases diagnosed prior to the 2021 World Health Organization (WHO) Classification of Tumors of the Central Nervous System (CNS) were retrospectively re-evaluated; consequently, only those meeting the current criteria for IDH-wildtype, CNS WHO grade 4 GB were included [28]. Furthermore, patients in the SBM group were required to present with a solitary intracranial lesion on the index MRI. Finally, the availability of comprehensive follow-up data sufficient for the calculation of OS was mandatory for inclusion.

To ensure the integrity of the volumetric and survival analyses, several exclusion criteria were strictly applied. Patients presenting with multiple simultaneous intracranial lesions were excluded from the SBM cohort. To avoid confounding the baseline imaging features, any individual who had received intracranial surgical intervention, radiotherapy, or systemic chemotherapy prior to the index MRI acquisition was also excluded from the study. Additionally, cases characterized by insufficient image quality that precluded reliable automated AI-based segmentation were omitted from the final dataset. The study further excluded patients lacking definitive histopathological confirmation or those with incomplete clinical and survival records, ensuring that only high-quality, verifiable data were utilized for predictive modeling.

### 2.3. MRI Acquisition Protocol

All MRI examinations were performed on a 1.5 Tesla General Electric Signa Explorer scanner (GE Medical Systems, Waukesha, WI, USA) using a standardized brain imaging protocol. Three volumetric sequences were acquired for each patient and subsequently used as input for the AI-based segmentation pipeline:

▪ 3D T2-weighted Fluid Attenuated Inversion Recovery (FLAIR): repetition time (TR) 7000 ms, maximum echo time (TE) (approximately 120 ms), slice thickness 1.8 mm, matrix 256 × 256, freq. FOV 25.6 cm, spacing 0 mm (contiguous), variable flip angle, NEX 1.00.
▪ 3D T1-weighted (3D T1W): TR 7.4 ms, TE 2.9 ms, slice thickness 2.0 mm, matrix 256 × 256, freq. FOV 25.6, spacing 0 mm (contiguous), flip angle 12°, NEX 1.00.
▪ 3D Fast Spoiled Gradient Echo T1-weighted (FSPGR, post-contrast): TR 10.0 ms, TE 4.2 ms, slice thickness 1.8 mm, matrix 288 × 224, freq. FOV 25.6, spacing 0 mm (contiguous), flip angle 12°, NEX 1.00. A gadolinium-based contrast agent was administered intravenously at a standard dose of 0.1 mmol/kg body weight prior to this acquisition.

The three sequences were acquired within the same imaging session for all patients, ensuring temporal consistency between pre- and post-contrast volumes.

### 2.4. AI-Based Automated Segmentation

#### 2.4.1. Segmentation Software

Automated tumor segmentation was performed using mdbrain version 2.7 (mediaire GmbH, Berlin, Germany; distributed in Romania via Supermedical), a Class IIa CE-certified medical device software [40,41,42,43].

The segmentation engine integrates a DL algorithm based on a three-dimensional CNN with a U-Net architecture, designed for volumetric brain lesion segmentation from multiparametric MRI input. The software ingested the three acquired sequences—3D T2 FLAIR, 3D T1W pre-contrast, and 3D T1W post-contrast FSPGR—as a co-registered, multi-channel input for inference.

#### 2.4.2. Segmentation Output Parameters

**Figure 2:**
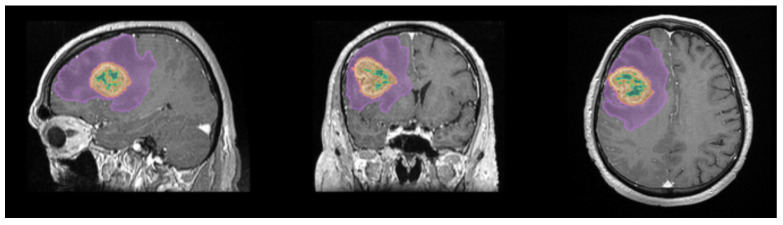
Mdbrain software processing of MRI brain acquisition (DICOM): the segmented regions include the necrotic core (green), the contrast-enhancing tumor component (orange), and the perilesional edema (purple) (approval was obtained from the Ethics Committee of the University of Medicine and Pharmacy “Grigore T. Popa” Iasi).

For each patient, the algorithm automatically delineated and quantified four distinct tumor subregions, yielding the following volumetric parameters:

- AI Model Total Volume: the aggregate volume of all segmented intratumoral compartments, expressed in cm^3^;
- AI Model Edema Volume: the volume of T2/FLAIR hyperintense signal surrounding the lesion, reflecting vasogenic edema extent;
- AI Model Necrosis Volume: the volume of the non-enhancing, centrally necrotic component;
- AI Model Contrast Volume: the volume of the gadolinium-enhancing rim, corresponding to the viable, highly vascularized tumor tissue (Figure 2).

These parameters were used as quantitative imaging biomarkers for both between-group differential analysis and survival correlation.

#### 2.4.3. Segmentation Validation

To assess the accuracy of the automated segmentation pipeline, AI-generated total tumor volumes were compared against manual reference measurements performed independently by two board-certified neuroradiologists with over 10 years of experience in neuro-oncological imaging.

Manual volumetry was conducted on the post-contrast T1W sequence using the ellipsoidal method. The tumor volume was determined by measuring the three longest orthogonal diameters (*A*, *B*, and *C*) in centimeters, and the final volume was calculated using the formula:

$$V=\frac{\pi }{6}\times \;A\;\times \;B\;\times \;C$$

The agreement between the AI-derived volumes and the manual reference standard (the mean of the two radiologists’ measurements) was assessed using Pearson’s correlation coefficient to evaluate potential systematic bias between the automated and manual methods [40,41,43].

### 2.5. Feature Engineering

Tumor localization was mapped to two structured categorical variables—a binary general localization (supratentorial vs. infratentorial) and a precise anatomical region. Tumor laterality was encoded as left, right, or center, and primary tumor histology was standardized to five classes (GB, breast, melanoma, pulmonary, digestive), from which a binary diagnosis variable (GB vs. SBM) was subsequently derived.

To capture the relative tumor architecture independently of absolute size, three normalized volumetric ratios were computed from the AI-segmented compartments:

- AI Model Contrast Volume/AI Model Total Volume Ratio;
- AI Model Necrosis Volume/AI Model Total Volume Ratio;
- AI Model Edema Volume/AI Model Total Volume Ratio [44].

### 2.6. Survival Data and Prognostic Factors

OS was determined based on the interval from histopathological diagnosis to death, with 1 April 2025 serving as a proxy for missing mortality data. The use of this placeholder did not bias the statistical outcomes; censored observations were correctly flagged, and the analysis accounted for the true duration of the follow-up period. OS distributions were estimated non-parametrically using the Kaplan–Meier (KM) method [45]. Continuous variables, including patient age, manually estimated volumes, and all AI-derived volumetric compartments, were dichotomized at the cohort median for KM stratification.

Independent predictors of mortality were identified by fitting univariate Cox Proportional Hazards (CPHs) models. For each candidate variable, the CPH model yielded the Hazard Ratio (HR) with a 95% confidence interval (CI) and a *p*-value. Variables reaching *p* < 0.05 in the univariate analysis were considered significant prognostic factors and were further evaluated in the predictive modeling phase.

### 2.7. Statistical Analysis

Baseline characteristics were stratified by diagnosis (GB vs. SBM) and summarized as counts with percentages for categorical variables and as means with standard deviations or medians with interquartile ranges for continuous variables, depending on the distribution. Between-group comparisons of continuous measurements (manual volume, AI-derived total, contrast-enhancing, necrotic, and edema volumes) were performed using the non-parametric Mann–Whitney U test, as appropriate. Categorical variables were compared using the Pearson’s Chi-squared test or Fisher’s exact test. The agreement between the manual total tumor volumes and the AI-generated total volumes was quantified using Pearson’s correlation coefficient (r). Statistical significance was set at *p* < 0.05 [46].

### 2.8. Predictive Modeling

Six data-driven exclusion criteria were applied sequentially to remove cases with significant segmentation artifacts or biologically implausible values (e.g., AI Model Edema Volume/AI Model Total Volume Ratio ≥ 100%, or AI Model Contrast Volume ≥ 200 ${cm}^{3}$ where visual inspection confirmed “leakage” into dural sinuses). After exclusions, the analysis-ready dataset comprised 115 patients with complete volumetric and survival data.

A binary classification task was defined to predict survival: patients were labeled as short survivors (OS ≤ 6 months, *n* = 40) or long survivors (OS > 6 months, *n* = 75). Patients who were alive at the last follow-up but lacked a recorded date of death were confirmed to have survived beyond 6 months based on their most recent follow-up record and were therefore assigned to the corresponding survival category. To address the class imbalance (1.8:1), the scale_pos_weight parameter was optimized within the XGBoost 2.1.4 framework [47].

Three successive XGBoost classifier models were developed:

Model 1—Manual volumetry (8 features): Clinical/demographic variables plus the manual tumor volume.

Model 2—AI Volumetry (11 features): Clinical/demographic variables plus the four AI-segmented compartments (total, contrast-enhancing, necrotic, and edema volumes).

Model 3—Enhanced feature model (14 features): Model 2 features plus the three normalized radiomic ratios (AI Model Contrast Volume/AI Model Total Volume Ratio, AI Model Necrosis Volume/AI Model Total Volume Ratio, and AI Model Edema Volume/AI Model Total Volume Ratio).

Given the limited sample size, a nested cross-validation strategy was employed to ensure model stability and prevent overfitting. Specifically, model performance was assessed using stratified 5-fold cross-validation in the outer loop, while hyperparameter optimization was performed via grid search within an inner cross-validation loop. The tuned hyperparameters included the learning rate, maximum tree depth, and number of estimators. The best-performing model was obtained with a learning rate of 0.01, a maximum depth of 2, and 200 estimators. Model discrimination was quantified using: the mean Area Under the Receiver Operating Characteristic Curve (AUC-ROC) and its standard deviation, Precision Recall Area Under the Curve (PR-AUC), F1-score, sensitivity, and specificity. In the present study, class imbalance was addressed using model parameters; alternative strategies such as focal loss and Synthetic Minority Oversampling Technique (SMOTE) were not evaluated and remain to be investigated in future work. Feature importance was extracted using the feature_importances_attribute to identify the most relevant prognostic biomarkers [48].

### 2.9. Software, Packages, and Reproducibility

All statistical analyses and machine learning (ML) workflows were implemented in Python 3.13. The primary libraries used included pandas and numpy for data management, scipy.stats for inferential testing, lifelines for survival analysis, and scikit-learn alongside xgboost for predictive modeling and performance evaluation.

## 3. Results

### 3.1. Patient Demographics and Clinical Characteristics

The final study cohort consisted of 123 patients with a mean age at diagnosis of 59.3 years (range: 38.0–75.9 years). The population showed a male predominance, with 78 males (63.4%) and 45 females (36.6%). Histopathological confirmation identified 84 cases of GB (68.3%) and 39 cases of SBM (31.7%). Among the metastatic group, the primary tumor origins were predominantly pulmonary (19.5%), followed by breast (5.7%), digestive (4.1%), and melanoma (2.4%). The vast majority of lesions were supratentorial (91.1%), while 8.9% were infratentorial. The demographic and clinical parameters of the study population are delineated in Table 2.

### 3.2. Validation of AI-Based Automated Volumetry

The automated AI-based segmentation demonstrated near-perfect linear agreement with manual neuroradiologist estimates, yielding a Pearson correlation coefficient of r = 0.955 (*p* < 0.001). The mean Manual Volume was 33.61 cm^3^, while the mean AI Model Total Volume was slightly higher at 37.83 cm^3^, resulting in a mean Manual/AI Model Total Volume Ratio of 0.87. This suggests a systematic, though minor, underestimation by manual ellipsoidal measurements compared to the more granular volumetric segmentation provided by the AI. Beyond the total volume, the AI successfully quantified sub-compartments unavailable to standard manual methods: a mean AI Model Contrast Volume of 25.65 cm^3^, a necrotic core of 12.64 cm^3^, and an AI Model Edema Volume of 79.77 cm^3^.

### 3.3. Comparative Volumetric Analysis: GB vs. SBM

**Figure 3:**
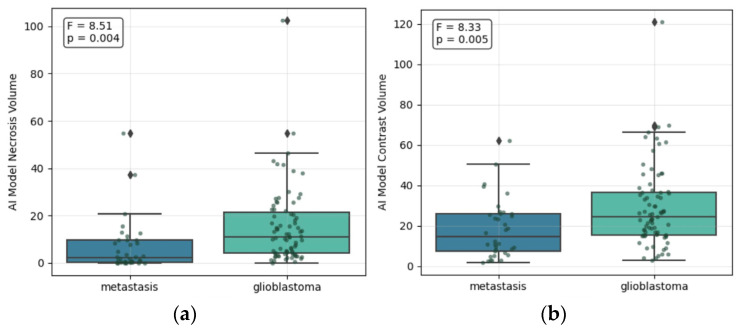
SBM vs. GB: (**a**) Statistical difference in the AI Model Necrosis Volume; (**b**) Statistical difference in the AI Model Contrast Volume.

Mann–Whitney U testing revealed significant differences in tumor architecture between the two groups:

- Necrosis: GB exhibited a significantly higher necrotic fraction than SBM (*p* < 0.001), with a mean AI Model Necrosis Volume/AI Model Total Volume Ratio of 0.28.
- Contrast enhancement: SBM demonstrated a significantly higher AI Model Contrast Volume/AI Model Total Volume Ratio (*p* < 0.001), reflecting a more homogeneous enhancement pattern (Figure 3).
- Peritumoral edema: While absolute edema volume did not differ significantly (*p* = 0.798), the AI Model Edema Volume/AI Model Total Volume Ratio was significantly higher in SBM (*p* = 0.003), indicating that the relative edema burden is a superior diagnostic discriminator (see Table 3).

Correlation and ANOVA analyses revealed a consistent pattern of associations between clinical, anatomical, and volumetric features. Age at diagnosis was inversely correlated with AI Model Total Volume (r = −0.201, *p* = 0.031) and AI Model Necrosis Volume (r = −0.279, *p* = 0.003), and negatively associated with the AI Model Necrosis Volume/AI Model Total Volume Ratio (r = −0.201, *p* = 0.032), while showing a weak positive correlation with the AI Model Contrast Volume/AI Model Total Volume Ratio (r = 0.197, *p* = 0.035)—collectively suggesting that younger patients tend to present with larger, more necrotic tumors.

Tumor general localisation (supratentorial vs. infratentorial) was significantly associated with multiple volumetric compartments, most strongly with edema volume (F = 9.05, *p* = 0.003), AI Model Contrast Volume/AI Model Total Volume Ratio (F = 8.44, *p* = 0.004), and AI Model Necrosis Volume/AI Model Total Volume Ratio (F = 7.94, *p* = 0.006), indicating that tumor compartment composition differs systematically by anatomical compartment.

Primary tumor histology was the most broadly associated categorical variable, showing significant relationships with age at diagnosis (F = 3.11, *p* = 0.018), AI Model Total Volume (F = 3.14, *p* = 0.017), and all three normalized ratios, with the strongest effect observed for the AI Model Edema Volume/AI Model Total Volume Ratio (F = 6.82, *p* < 0.001).

**Figure 4:**
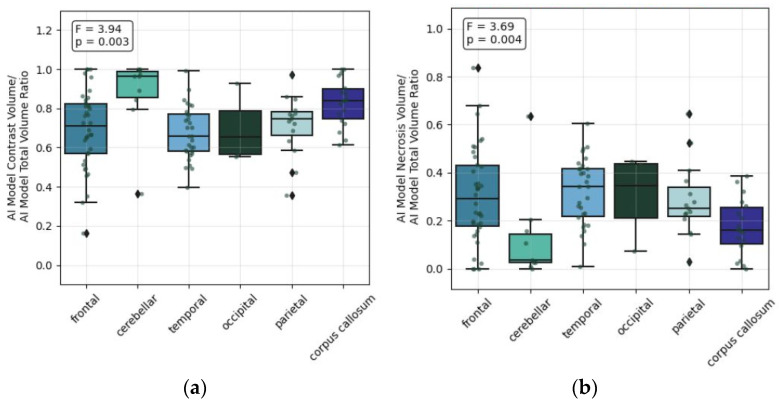
Anatomical localization: (**a**) Statistical difference in the AI Model Contrast Volume/AI Model Total Volume Ratio; (**b**) Statistical difference in the AI Model Necrosis Volume/AI Model Total Volume Ratio.

Precise anatomical localization was similarly associated with total tumor volume, necrotic and edema volumes, and both the AI Model Contrast Volume/AI Model Total Volume Ratio (F = 3.94, *p* = 0.003) and AI Model Necrosis Volume/AI Model Total Volume Ratio (F = 3.69, *p* = 0.004) ratios, reinforcing the notion that tumour biology and compartment composition are substantially shaped by the specific anatomical site of involvement (Figure 4).

### 3.4. Survival Analysis Results

**Figure 5:**
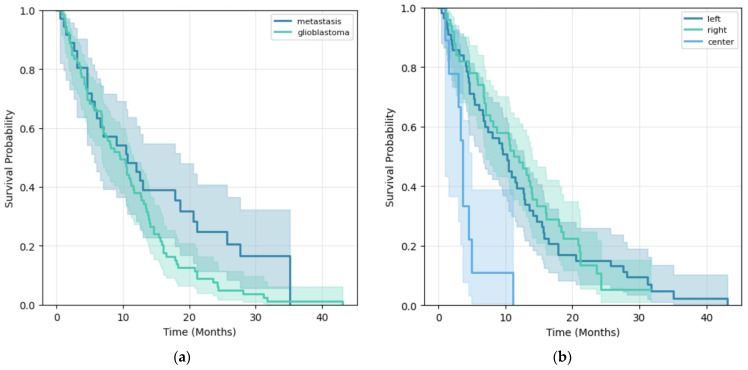
Kaplan–Meier survival curves: (**a**) Glioblastoma vs. Single Brain Metastasis; (**b**) Tumor laterality.

Kaplan–Meier analysis revealed an overall median OS of 10.66 months. While survival curves favored SBM patients, the difference only reached a trend toward significance (log-rank *p* = 0.062). Interestingly, a significant clinical predictor in KM analysis was tumor laterality (*p* < 0.001), with central tumors showing lower OS (Figure 5). After encoding laterality as a binary variable for the Cox Proportional Hazards model, tumor side remained an independent prognostic factor (HR = 0.685, 95% CI: 0.481–0.975, *p* = 0.0359).

Univariate Cox Proportional Hazards regression identified AI-derived Contrast Volume as a significant independent predictor of mortality (HR > 1, *p* < 0.05), meaning larger contrast cores were associated with a higher risk of death. Notably, neither manual volume nor total AI volume reached significance independently, suggesting that internal compartment data carries more prognostic weight than gross tumor size.

### 3.5. Classification ML Model Performance (6-Month Survival)

**Figure 6:**
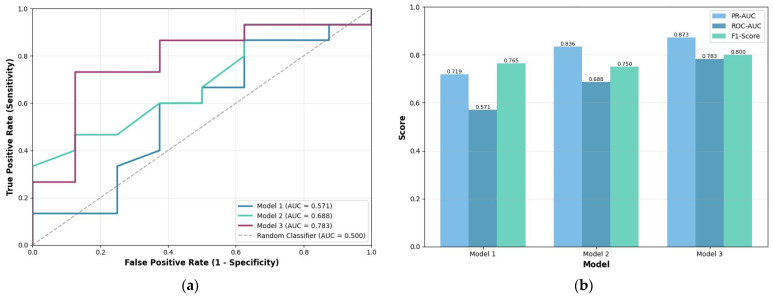
Model test sets metrics: (**a**) ROC curves comparison; (**b**) PR-AUC, ROC-AUC, and F1-Score comparison.

After applying data-driven exclusions, 115 patients were used for the predictive modeling task. The enhanced feature model (Model 3), which integrated clinical data with AI-derived volumetric ratios, outperformed both the Manual and AI-Volumes-only models. Also, the sensitivity of the models was: 0.866 (Model 1), 0.800 (Model 2), and 0.933 (Model 3) (Figure 6).

Discrimination performance, quantified by the ROC-AUC, was highest for Model 3 on the test set (ROC-AUC = 0.783), followed by Model 2 (0.688) and Model 1 (0.571). Cross-validation analysis confirmed the superior performance of Model 3, with a mean ROC-AUC of 0.713 ± 0.180, compared to 0.682 ± 0.117 for Model 1 and 0.675 ± 0.112 for Model 2. The enhanced model represented a +21.2% improvement over manual volumetry. However, despite this gain in discrimination, the specificity of all three models remained low (Model 1: 0.250; Model 2: 0.375; Model 3: 0.250), indicating a high false-positive rate. The improvement should therefore be interpreted as exploratory evidence that relative tumor composition may carry additional prognostic information beyond gross size, rather than as confirmation of clinical readiness.

### 3.6. Feature Importance Analysis

The top predictors in the enhanced model (Model 3) were both AI-derived normalized ratios: the AI Model Contrast Volume/AI Model Total Volume Ratio (0.224) and the AI Model Edema Volume/AI Model Total Volume Ratio (0.143), followed by total volume and laterality.

Diagnosis type (GB vs. SBM) contributed minimally to this model (importance approx 0.02), suggesting that the AI-derived architectural features effectively “captured” the biological differences between the two pathologies within the survival prediction framework.

## 4. Discussion

The primary objective of this study was to determine if AI-based MRI segmentation could “bridge the gap” between standard radiological imaging and clinical survival outcomes in patients with GB and SBM. Our exploratory results suggest that, while manual volumetry provides a basic estimate of tumor size, AI-derived sub-compartment analysis and normalized volumetric ratios may offer improved diagnostic discrimination and prognostic accuracy in this preliminary cohort.

### 4.1. Diagnostic Differentiation Through AI Sub-Compartments

The automated segmentation pipeline showed strong volumetric correlation with manual neuroradiologist measurements, as reflected by a Pearson correlation coefficient of r = 0.955 (*p* < 0.001), a level of concordance consistent with previously reported validation studies of CNN-based U-Net architectures applied to brain tumor segmentation [41,43]. The observed systematic overestimation of tumor volume by the AI relative to the ellipsoidal manual method (mean Manual/AI Model Total Volume Ratio of 0.87) is a well-recognized phenomenon attributable to the inherent geometric approximation introduced by the ellipsoidal formula, which tends to underestimate irregular tumor contours. The volumetric superiority of voxel-based DL segmentation over simplified geometric models has been consistently reported in the neuro-oncological imaging literature [36,49,50,51]. Crucially, the AI tool provided granular sub-compartmental quantification—including contrast-enhancing tissue, necrotic core, and peritumoral edema—measurements that are practically inaccessible through routine clinical methods, thereby establishing the methodological foundation for the subsequent differential and prognostic analyses.

The comparative volumetric analysis revealed statistically significant differences in tumor architecture between GB and SBM cohorts across multiple compartments, corroborating the distinct biological behaviors of these two entities as described in the existing literature. GB exhibited significantly greater absolute volumes alongside a markedly higher necrotic fraction, consistent with its infiltrative, hypoxia-driven growth pattern and the pseudo-palisading necrosis that constitutes one of its histopathological hallmarks [52,53]. Conversely, SBM demonstrated a significantly higher contrast-enhancing fraction and a greater relative edema burden—findings that align with the well-established tendency of metastatic lesions to form a compact, highly vascularized tumor mass that exerts disproportionate vasogenic edema relative to its core volume [41,54].

Notably, while absolute peritumoral edema volumes did not differ significantly between the two groups (*p* = 0.798), the AI Model Edema Volume/AI Model Total Volume Ratio was significantly elevated in SBM (*p* = 0.003). This dissociation underscores a critical methodological insight: raw volumetric measurements may be confounded by inter-individual variation in absolute tumor burden, whereas normalized architectural ratios more faithfully capture the underlying biological phenotype. This principle—that relative compartment composition is a superior discriminator than absolute size—has important implications for radiological practice, where the edema-to-lesion ratio has previously been proposed as a qualitative differential criterion but has rarely been quantified in a systematic, automated fashion [55,56].

**Figure 7:**
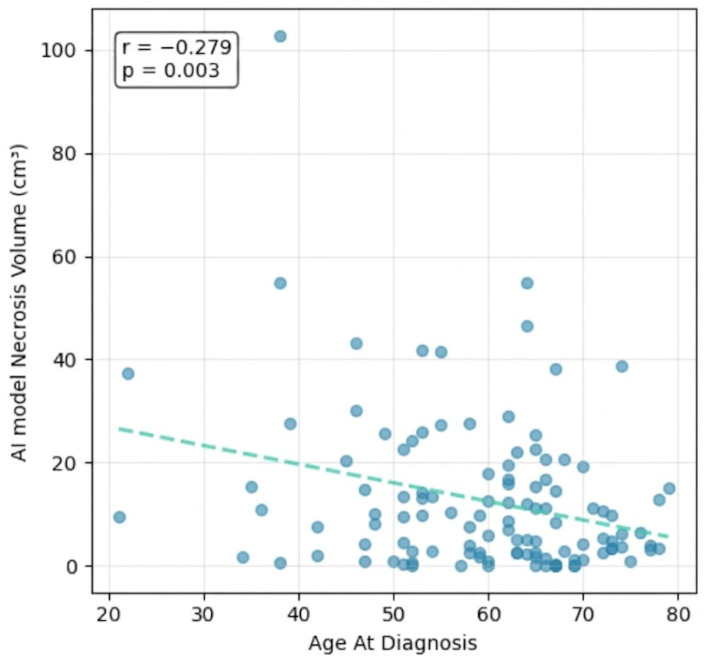
Age vs. AI Model Necrosis Volume correlation.

The inverse correlation between age at diagnosis and both AI Model Total Volume and AI Model Necrosis Volume (r = −0.201, *p* = 0.031, and r = −0.279, *p* = 0.003, respectively) represents a noteworthy secondary finding (Figure 7). Younger patients tended to present with larger, more necrotic tumors, a pattern that may reflect biological differences in tumor aggressiveness across age groups, differential immune microenvironmental responses, or a longer symptomatic interval before diagnosis in younger individuals who may initially attribute neurological symptoms to non-neoplastic causes.

The significant association between anatomical localization and compartment composition—particularly the strong relationship between infratentorial location and edema volume (F = 9.05, *p* = 0.003)—further reinforces the notion that tumor biology is partially shaped by the neurovascular and microenvironmental characteristics of the specific anatomical compartment [57,58].

### 4.2. Bridging the Gap to Survival Prediction

Our study highlights a clear hierarchy in predictive modeling for 6-month survival. Standard clinical variables combined with manual volumetry yielded a poor AUC of 0.571, indicating that gross tumor size alone is a weak predictor of mortality. The transition to multi-compartment AI volumes (Model 2) improved performance to 0.688, while the highest accuracy in this exploratory analysis was observed for the enhanced feature model (Model 3), with an AUC of 0.783. Given the limited sample size and the absence of external validation, this incremental gain should be interpreted as preliminary.

The inclusion of normalized ratios—specifically the AI Model Contrast Volume/AI Model Total Volume Ratio and AI Model Edema Volume/AI Model Total Volume Ratio—appeared to be the primary driver of this 21.2% improvement over manual methods within the present cohort. This may suggest that how a tumor is “built” (the ratio of viable tissue to necrosis and surrounding edema) could be more prognostically informative than its absolute dimensions. Furthermore, the fact that the diagnosis (GB vs. SBM) contributed minimally to the enhanced model’s importance (0.02) is compatible with the hypothesis that these AI-derived compositional features partially capture biological behavior across both tumor types, although this interpretation requires confirmation in larger, molecularly characterized cohorts.

### 4.3. Methodological Positioning Relative to End-to-End Approaches

While our framework leverages a clinically deployable, CE-certified DL segmentation tool (mdbrain, a U-Net-based 3D CNN) coupled with gradient-boosted classifiers operating on interpretable, biologically meaningful volumetric ratios, an alternative line of research has explored fully end-to-end DL architectures and, more recently, foundation models that jointly learn representations and prognostic outputs directly from raw imaging data [59]. Such approaches benefit from increasingly expressive segmentation backbones—ranging from U-Net variants to recent E-shaped and transformer-based designs [60]—and from large-scale pretraining paradigms that mitigate annotation scarcity and enable task-agnostic transfer across imaging modalities [59].

However, these end-to-end paradigms typically demand substantially larger, well-curated training cohorts, often behave as black boxes, and remain difficult to integrate into routine clinical workflows where transparent, auditable risk attribution is required. In parallel, the real-time integration of AI directly with MRI acquisition hardware has been shown to reduce inter-operator variability and improve the reproducibility of downstream analyses [61], a complementary direction that could further standardize the imaging inputs feeding prognostic models such as ours.

Our hybrid design combines a regulatory-approved DL segmenter with a compact, interpretable set of normalized sub-compartment ratios. This architecture prioritizes clinical applicability and prognostic transparency, particularly under limited sample sizes. Importantly, our approach remains methodologically compatible with the broader trend toward fully end-to-end, foundation-model-based neuro-oncological pipelines, rather than competing against it.

### 4.4. Clinical and Biological Implications

The present analysis suggests a possible trend toward reduced overall survival in patients with centrally located tumors; however, this observation requires confirmation in larger cohorts. This may reflect the increased surgical complexity and higher risk of involving critical midline structures. Additionally, the finding that contrast volume emerged as a significant independent predictor of mortality in Cox regression (*p* < 0.05) is consistent with the prevailing biological view that extensive contrast enhancement may be associated with treatment resistance and more rapid progression, although a causal interpretation is beyond the scope of the present exploratory analysis.

From a surgical planning perspective, the identification of contrast-enhancing volume as an independent prognostic factor is consistent with the oncological rationale for maximal safe resection targeting the enhancing compartment, as has been advocated in both GB and resectable brain metastasis management guidelines [62,63]. The observation that centrally located tumors were associated with inferior survival in this cohort suggests that AI-derived anatomical and volumetric data could, pending prospective validation, contribute to preoperative risk stratification models and support more nuanced counseling regarding surgical intent, expected functional outcomes, and overall prognosis.

### 4.5. Limitations and Future Directions

Several limitations of the present study warrant careful consideration. The retrospective single-center design and the cohort size represent an inherent constraint, limiting the generalizability of the findings. Prospective, multicenter validation across diverse MRI platforms, field strengths, and patient populations is required before these results can inform routine clinical practice.

An additional clinically important limitation concerns the predictive performance of the survival models. Although the enhanced feature model (Model 3) achieved a higher AUC-ROC than the manual volumetry baseline, the specificity of all three models remained low. This indicates a high false-positive rate, meaning the models tend to overcall short-term mortality. A predictor with high sensitivity but low specificity has limited standalone clinical utility and cannot, in its current form, serve as a decision-support tool. The models should therefore be regarded as exploratory hypothesis-generating tools rather than as validated clinical instruments. Improving specificity will likely require larger and more balanced training cohorts and prospective recalibration on external data.

A further methodological limitation relates to the validation of the AI-based segmentation. Although AI-derived total volumes showed strong linear correlation with manual ellipsoidal estimates (r = 0.955, *p* < 0.001), correlation does not fully capture agreement, as it is insensitive to systematic bias and proportional differences. No voxel-wise validation against manual reference segmentations and no formal agreement analysis (such as Bland–Altman analysis or Dice similarity coefficients) were performed in the present cohort. The validation reported here should therefore be interpreted as a consistency check between two volumetric methods rather than as a formal accuracy assessment of the segmentation tool itself.

Furthermore, the integration of molecular biomarkers—such as IDH mutation status, MGMT promoter methylation, EGFR amplification, and tumor mutational burden—into the predictive modeling framework represents a scientifically warranted extension of the current approach, given the established prognostic weight of these variables in both primary and secondary brain tumors. Importantly, in the absence of such molecular validation, the imaging-based biomarkers proposed here cannot be considered surrogates for, or alternatives to, established molecular predictors; rather, they should be regarded as candidate complementary features whose true incremental value can only be assessed once integrated multimodal (imaging plus molecular) datasets become available. The clinical applicability of the present findings is therefore inherently limited, and the conclusions drawn from this work should be understood strictly within this exploratory framework.

Addressing these limitations in future prospective, multi-institutional studies will be essential for establishing AI-driven volumetric segmentation as a validated, clinically deployable tool in the neuro-oncological diagnostic and prognostic workflow.

## 5. Conclusions

Our study suggests that, in this exploratory cohort, AI-based automated segmentation showed strong volumetric concordance with manual neuroradiologist estimates (r = 0.955) and may serve as a useful complementary tool for characterizing the complex internal architecture of intracranial lesions. By quantifying distinct tumor sub-compartments, the AI-driven pipeline identified that the relative composition of a tumor—specifically the Necrosis/Total and Edema/Total ratios—may represent promising imaging biomarkers that, in this exploratory cohort, appeared more informative than gross tumor size alone.

The integration of these AI-derived features into an ML framework resulted in an encouraging, albeit preliminary, improvement in predictive accuracy for 6-month OS, increasing the AUC-ROC from 0.571 (manual) to 0.783 (enhanced AI model). This finding supports the hypothesis that high-fidelity, automated radiomic features may help narrow the “gap” between static MRI and clinical outcomes, although these results require external validation before any definitive conclusion can be drawn.

Ultimately, the transition from scalar manual planimetry to multi-compartment AI segmentation may offer clinicians a more standardized and objective approach to lesion characterization. However, given the retrospective nature of this study, the limited sample size, and the absence of molecular biomarkers (such as IDH mutation status, MGMT promoter methylation, and EGFR amplification), these findings should be regarded as exploratory. Prospective, multicenter validation that integrates molecular profiling will be required before AI-based volumetry can be considered for routine implementation in neuro-oncological workflows.

As a summary, this study provides preliminary evidence that AI-assisted multi-compartment segmentation may represent a useful step forward in neuro-oncology imaging. By shifting the focus from gross tumor size to a more nuanced analysis of sub-compartment ratios, AI-based volumetry could help complement conventional MRI assessment in the prognostic evaluation of GB and SBM, pending confirmation in larger, prospective cohorts.

## Figures and Tables

**Table 1:** Differences in the epidemiological characteristics of GB and SBM.

Characteristic	GB	SBM	References
**Incidence**	~3.2–5.0 per 100,000 person-years	~10.0 per 100,000 person-years (overall BM), with SBM accounting for 30–50% of these cases	[9,10,11]
**Prevalence**	Most common primary malignant brain tumor (~48% of primary malignancies)	Most common overall malignant brain tumor; occurs in 20–40% of all systemic cancer patients	[9,12,13]
**Median age at diagnosis**	64 years (peak incidence between 55 and 74 years)	58–60 years (highly dependent on the primary cancer type)	[14,15]
**Sex distribution** **(M:F)**	1.6:1 (significant male predominance)	Varies by primary (e.g., lung/melanoma higher in M; breast higher in F) overall ~1.2:1	[14,16]
**Common locations**	Supratentorial (F and T lobes) Rarely crosses the midline via the corpus callosum	Gray-white matter junction; often distributed based on blood flow (80% supratentorial, 15% cerebellum)	[15,17]
**Growth pattern**	Infiltrative: microscopic tumor cells extend far beyond the visible MRI enhancement	Expansile: usually well-circumscribed; pushes rather than infiltrates surrounding brain tissue	[18,19]
**Known risk factors**	Ionizing radiation; specific genetic syndromes (e.g., Li-Fraumeni, Lynch)	History of systemic malignancy (lung, breast, renal, melanoma, colorectal)	[20,21]
**Prognosis** **(median OS)**	~12–15 months (standard of care) <10% 5-year survival	~6–12 months (variable; depends on “graded prognostic assessment” and systemic control)	[22,23]

BM: brain metastases; F: frontal; GB: glioblastoma; M:F: Male-to-Female ratio; OS: overall survival; SBM: single brain metastasis; T: temporal.

**Table 2:** The descriptive parameters pertaining to the study population.

Characteristics	Categories/Measure	No. of Patients	Average Value	Range (5–95%)
Sex	M	78	63.4%	N/A
	F	45	36.6%	N/A
Age at diagnosis (years)	Mean	123	59.32	38.00–75.90
Tumor type	GB	84	68.3%	N/A
	SBM	39	31.7%	N/A
Primary tumor	GB	84	68.3%	N/A
	Pulmonary	24	19.5%	N/A
	Breast	7	5.7%	N/A
	Digestive	5	4.1%	N/A
	Melanoma	3	2.4%	N/A
Tumor localization	Supratentorial	112	91.1%	N/A
	Infratentorial	11	8.9%	N/A
	Left	62	50.4%	N/A
	Right	51	41.5%	N/A
	Center	10	8.1%	N/A
**Tumor measurements**				
Manual Volume (cm^3^)	Mean	123	33.61	3.43–86.80
AI Model Total Volume (cm^3^)	Mean	123	37.83	4.91–87.91
AI Model Contrast Volume (cm^3^)	Mean	123	25.65	3.70–62.52
AI Model Necrosis Volume (cm^3^)	Mean	123	12.64	0.03–41.63
AI Model Edema Volume (cm^3^)	Mean	123	79.77	18.01–169.14
Manual/AI Model Total Volume Ratio	Mean	123	0.87	0.51–1.22
AI Model Contrast Volume/AI Model Total Volume Ratio	Mean	123	0.72	0.43–1.00
AI Model Necrosis Volume/AI Model Total Volume Ratio	Mean	123	0.26	0.01–0.56
AI Model Edema Volume/AI Model Total Volume Ratio	Mean	123	4.12	0.32–9.13
OS (months)	Mean	123	10.66	1.31–27.01

F: female; GB: glioblastoma; M: male; N/A: not applicable; OS: overall survival; SBM: single brain metastasis.

**Table 3:** Mann–Whitney U test results for volumetric differences between GB and SBM cohorts.

Volumetric Feature	GB vs. SBM (*p* Value)	Interpretation
Manual Volume	*p* = 0.005	GB significantly larger
AI Model Total Volume	*p* < 0.001	GB significantly larger
AI Model Contrast Volume	*p* = 0.002	GB significantly larger
AI Model Necrosis Volume	*p* < 0.001	GB is significantly more necrotic
AI Model Edema Volume	*p* = 0.798	No significant difference
AI Model Contrast Volume/AI Model Total Volume Ratio	*p* < 0.001	SBM has a higher contrast fraction
AI Model Necrosis Volume/AI Model Total Volume Ratio	*p* < 0.001	GB has a higher necrotic fraction
AI Model Edema Volume/AI Model Total Volume Ratio	*p* = 0.003	SBM has a higher relative edema burden

AI: artificial intelligence; GB: glioblastoma; SBM: single brain metastasis.

## Data Availability

The data presented in this study are available on request from the corresponding author due to ethical restrictions.
